# Correlation analysis of combined layers in multiplex networks based on entropy

**DOI:** 10.1371/journal.pone.0276344

**Published:** 2022-10-28

**Authors:** Dan Wang, Feng Tian, Daijun Wei

**Affiliations:** School of Mathematics and Statistics, Hubei Minzu University, Enshi, Hubei, China; University Campus Bio-Medico of Rome, ITALY

## Abstract

The interactions between layers of a multiplex network would generate new structural features, the most prominent feature being the existence of link overlaps between layers. How to capture the associations with the network behavior through the structural interaction between the combined layers of the multiplex network is a critical issue. In this paper, a new structure entropy is proposed by combining the overlapping links between the combined layers of a multiplex network. The correlation between layers is evaluated by structure entropy, and the results are consistent with the behaviors exhibited by the network. In addition, the validity and applicability of the proposed method were verified by conducting trials on four sets of real multiplex network data, which included the multiplex social network of a research department at Aarhus, tailor shop multiplex network, C. elegans multiplex network, and the network collected by Vickers from 29 seventh grade students in a school in Victoria.

## 1 Introduction

As the properties of complex networks have been studied in-depth, researchers have found that a simple network structure cannot accurately describe the interactions between individuals of complex systems [1–3]. In fact, the topology of a simple network alone is not sufficient to describe the properties of various complex systems, and multiple layers need to be considered to describe the interactions between different networks [4–6]. In multilayer networks, layers tend to interact and influence each other through different ways [7, 8]. That is, there are many different types of layers between the same individuals in the network, and each layer corresponds to a different type of interaction. For example, in a multilayer transportation network [9, 10], people can reach the same location through different transportation modes.

There is growing experimental evidence that the network structure of real systems behaves as a multilayer network, and the most common of the multilayer networks is the multiplex network [11]. In multiplex networks, a set of nodes can belong to multiple network layers at the same time, and have different connection methods at different layers [12]. For example, in a power network and a communication network [1], the same group of nodes has both a power supply relationship and a communication control relationship. A salient feature of multiplex networks is the interlayer correlation, that is, two nodes that are connected in one layer may also be connected in other layers [13]. This common correlation is also the overlapping edges between networks [14, 15]. When a node fails in a single transport layer, it cannot be traversed by any path. However, if this node is part of a multiplex network, it can still be reached at other layers. This inherent feature of multiplex networks enhances the flexibility of the system compared with single-layer networks.

The particular structural property of layers coupled to each other in multiplex networks brings many conveniences. For example, multiplex biological networks integrate different network layers that map the biological organization of our body at different levels, from genome to transcriptome, proteome and phenotype [16]. By mapping protein interactions and mechanisms, previously unknown proteins that act in disease can be better characterized, allowing for faster tracking of genetic defects. Therefore, the use of interlayer coupling to evaluate the correlation between layers of a multiplexed network and the relationship with network behavior is the focus of this paper. Although the interaction between layers results in interlayer correlations that are a prominent feature of all multiplex networks, there is no complete and general theoretical approach to describe and quantify the interlayer correlations.

Depending on the information theory, the structure entropy of complex networks can be used to measure the complexity of complex networks [17, 18]. Among the existing methods in this field, the vast majority are based on Shannon entropy, the degree and betweenness of nodes in the network quantify the structural complexity of complex networks in the form of entropy [19, 20]. For many weighted multiplex networks [21, 22], the overlapping links between different layers may also be correlated with link weights. The existing weighted multiplex network model based on maximum entropy [23] can be used to simulate the dynamic process on different network topologies and generate multiplex networks with different types [24–26]. Similar to the single-layer network, the maximum entropy theory of network structure can be used to evaluate the information content of the network structure [27, 28]. In this paper, the overlapping links between layers of a multiplex network are used to calculate the structure entropy of each combined layer, and the correlation and stability of each layer are analyzed by the network structure entropy.

In multiplex networks, the coupling between different layers makes the relationship of combined layers complicated. For example, a diplomatic dispute between two countries may lead to war, but it is worth considering which has the most influence in the dispute that leads to war. In other words, wars between countries and different diplomatic disputes can be treated as a multiplex network, so that the problem can be transformed into studying the correlation between the different layers (disputes) and the war outbreak layer or studying the stability between the pairwise. In addition, for the present many airlines, they compete and cooperate with each other, these different companies can be regarded as a multiplex network. The extent to which the decisions of one company affect the decisions of another is an issue worth studying, which is also equivalent to the question of researching the correlation between layers. Based on that, this paper proposes a network structure entropy to calculate the correlation of combined layers and describe the corresponding stability in multiplex networks. Through numerical simulations, and tests on four real multiplex networks, which include AUCS multiplex network, Tailor shop multiplex network, C. elegans multiplex network, and 7thGraders multiplex network. The experimental results are consistent with the behavior of the real networks, which also confirms the effectiveness and applicability of the proposed method.

The rest of the paper is organized as follows. In section 2, the definition of the multiplex network, the characteristics of overlapping links and their representation, and existing structure entropy are introduced. In section 3, the algorithm of this paper is introduced through a simple numerical simulation. In section 4, we show the application of our algorithm to four different types of multiplex networks and provide some interesting insights. Finally, some conclusions are summarized in section 5.

## 2 Preliminaries

This section describes the definition of the multiplex network, the characteristics of overlapping links and their representation, and existing structure entropy.

### 2.1 Multiplex network

#### 2.1.1 Definition of multiplex network

A multilayer network can be represented by a binary group *M* = (*G*, *C*) [4], where network *G* = {*G*^*α*^;*α* ∈ {1, 2, ⋯, *L*}} represents a set of (directed or undirected, weighted or unweighted) graphs *G*^*α*^ = (*V*^*α*^, *E*^*α*^). *G*^*α*^ is called layer *α* of the multilayer network *M*, $V^{\alpha }=\left\{v_{1}^{\alpha },v_{2}^{\alpha },\cdots ,V_{N^{\alpha }}^{\alpha }\right\}$ represents the node set of layer *G*^*α*^ (*N*^*α*^ is the number of nodes in layer *G*^*α*^), *E*^*α*^ represents the intralayer connections of layer *G*^*α*^. *C* = {*E*^*αβ*^ ∈ *V*^*α*^×*V*^*β*^;*α*, *β* ∈ {1, ⋯, *L*}, *α* ≠ *β*} is the set of interconnections between nodes of different layers *G*^*α*^ and *G*^*β*^, while *E*^*αβ*^ denotes a single interlayer link of layers *G*^*α*^ and *G*^*β*^.

Multiplex network is a type of network in which the multilayer network *M* considers both *G* and *C* tuples, mathematically represented as *M* = (*G*, *C*), satisfying *V*^1^ = ⋯ = *V*^*L*^ = *L*. Its intralayer couplings are denoted as *E*^*αβ*^ = {(*v*, *v*);*v* ∈ *V*}(1 ≤ *α* ≠ *β* ≤ *L*). The mapping network corresponding to the multiplex network is *aggr*(*M*) = (*V*^*M*^, *E*^*M*^), where

$$V^{M}=V$$

EM=(⋃α=1L{(viα,vjα);(viα,vjα)∈Eα})∪(⋃α,β=1α≠βL{(viα,viβ);vi∈V}) (1)

#### 2.1.2 Characteristics of multiplex network

**Fig 1:**
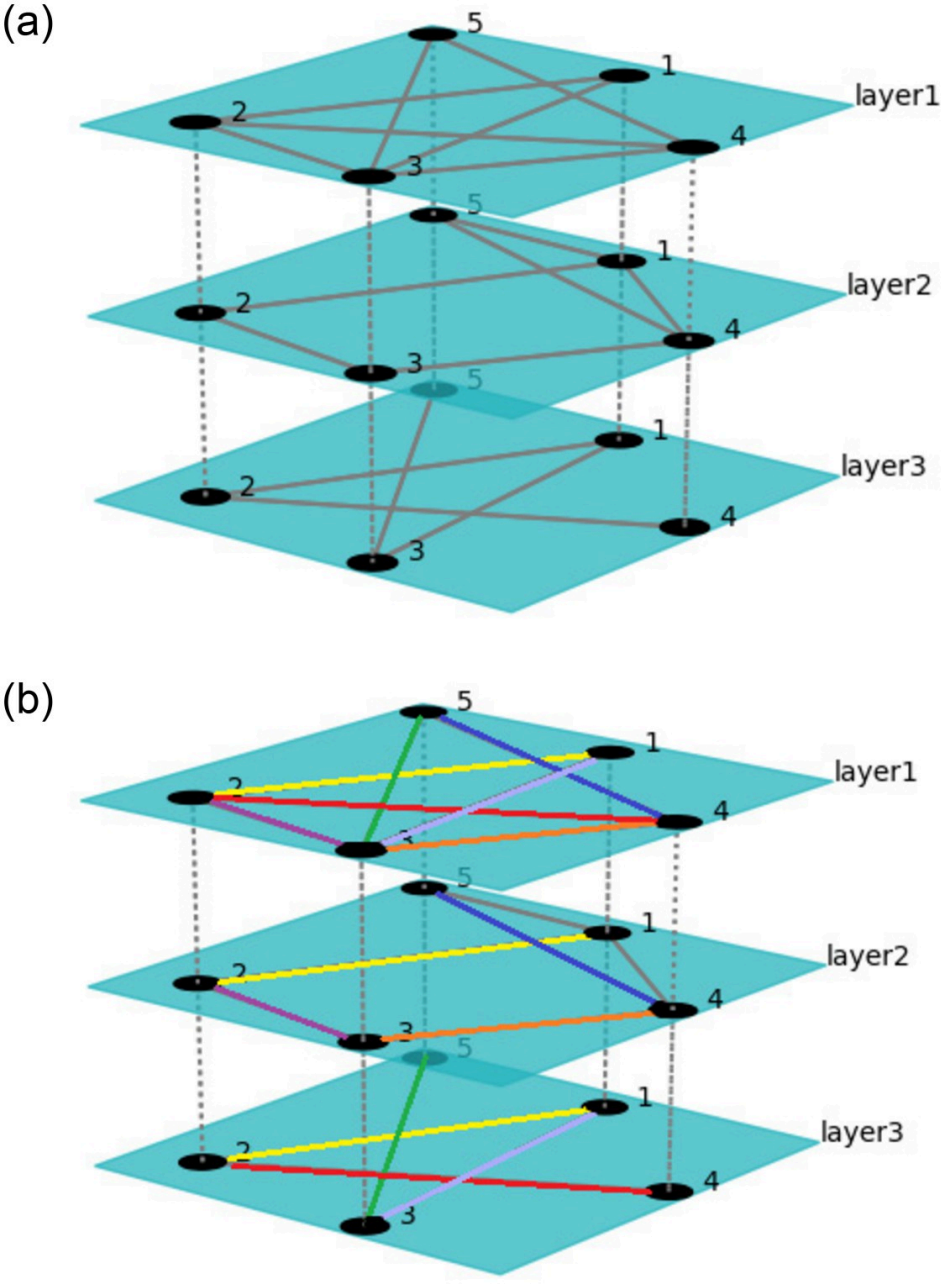
Schematic diagram of multiplex network structure with overlapping links at different layers. Fig 1(a) is a schematic diagram of a simple three-layer network, and the overlapping edges present in each network layer are marked in the same color in Fig 1(b).

A distinctive feature of multiplex network is that each layer has the same set of nodes but the edge attributes of each layer of nodes are different, and there are edges of the same node between layers. In other words, a multiplex network is a set of fixed nodes that are connected together by different types of links. A typical example of a multiplex network is a social system, which can be viewed as an overlay of multiple complex social networks in which people and connected edges represented between nodes can capture a variety of different social relationships. The network structure of this type is shown in Fig 1(a). The overlapping links of different layers in this network are marked with different colors in Fig 1(b), and the three-layer networks in the figure all show obvious overlapping links. This means that the number of overlapping links present in the different layers is extremely important for the global impact of the multiplex network. For example, in a collaborative citation network, two authors often cite each other’s papers in their papers, resulting in a significant overlap in the two-layer network.

### 2.2 Representation of overlapping links in multiplex networks: multilink

**Fig 2:**
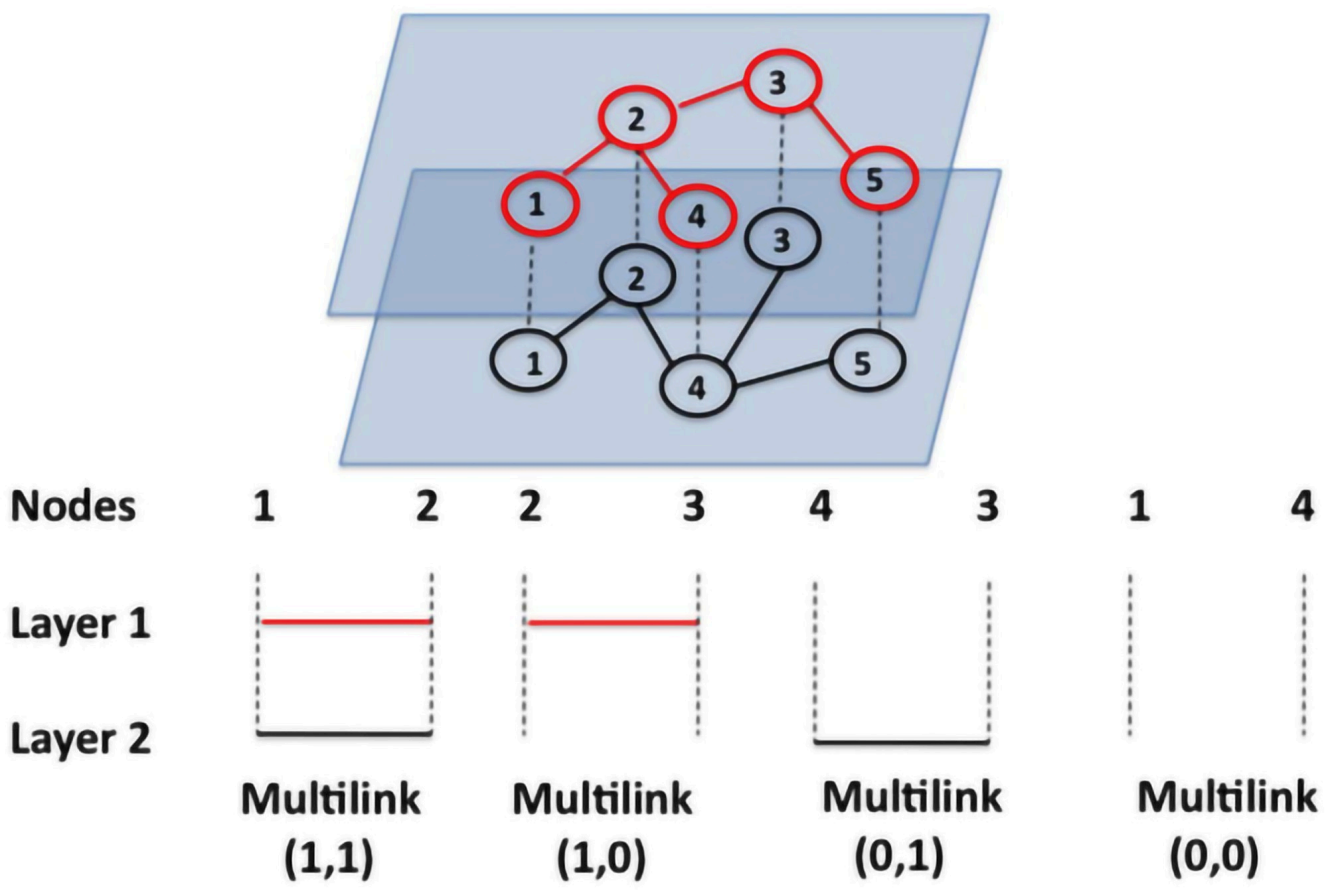
Example of all possible multilinks in a multiplex network with *M* = 2 layers and *N* = 5 nodes. Nodes *i* and *j* are linked by one multilink $\vec{m}=\left(m_{1},m_{2}\right)$. [21, 26]

The most straightforward way to describe network connections is multilink [21, 22, 26]. The multilink determines all connections between any two nodes in the multiplex network. Fig 2 illustrates a two-layer multiplex network, where each pair of nodes is connected by a given multilink [21, 26]. For a multiplex network, the multilink $\vec{m}=\left(m_{1},m_{2},\cdots ,m_{\alpha },\cdots ,m_{M}\right),m_{\alpha }=0,1$ is represented as a set of overlapping edge maps between nodes at different layers. In Fig 2, multilink(1,1) represents that node 1 and node 2 are connected by an edge in the first-layer network, and are also connected by an edge in the second-layer network. Therefore, the total number of multilink in the network is also the total number of node pairs that multilink are connected to. Based on multilink, the total number of multilink associated to a node can be defined as multidegree, expressed as

$$\begin{array}{c} k_{i}^{\vec{m}}=\sum_{j\neq i}A_{ij}^{\vec{m}},\;A_{ij}^{\vec{m}}=0,1 \end{array}$$ (2)

where $k_{i}^{\vec{m}}$ is the multilink of node *i*, and $A_{ij}^{\vec{m}}$ is the adjacency matrix of whether there are multilink between node *i* and node *j*.

### 2.3 Existing structure entropy

In information theory, Shannon entropy [29], is an uncertain measure of information in a system or process, which is denoted as *E*. Suppose *X* = {*x*_1_, *x*_2_, *x*_3_, ⋯, *x*_*n*_} is a discrete random variable, the appearance probability of information source given by *X* is denoted as *p*_*i*_, *i* = 1, 2, ⋯, *n* and $\sum_{i=1}^{X}p_{i}=1$. Then the information entropy is defined as follows,

$$\begin{array}{c} E=-\sum_{i=1}^{N}p_{i}\text{log}p_{i} \end{array}$$ (3)

as a generalization of Shannon entropy, Deng entropy is given as follows [30],

$$\begin{array}{c} E_{D}=-\sum_{i\subseteq X}p_{i}\text{log}\frac{p_{i}}{2^{\left|X\right|}-1} \end{array}$$ (4)

where *X* is the number of random variable, and *p*_*i*_ is the probability of state *i*.

Based on Deng entropy, a belief entropy is given in ref. [31] that can effectively discriminate information fusion situations, defined as follows,

$$\begin{array}{c} E_{W}=-\sum_{i\subseteq X}p_{i}\text{log}(\frac{p_{i}}{2^{\left|X\right|}-1}{\left(1+\varepsilon \right)}^{f(|X|)}) \end{array}$$ (5)

where *ε* is a constant and *ε* > 0. *f*|*X*| is the function about the cardinality of *X*.

Inspired by entropy, structure entropy is applied to measure the complexity of network. Many researchers have generalized methods to calculate the structure entropy of complex networks. Most of those structure entropies are based on the degree of the nodes [32, 33], defined as follows,

$$\begin{array}{c} E_{\text{deg}}=-\sum_{i=1}^{N}p_{i}\text{log}p_{i},\;p_{i}=\frac{Degree(i)}{\sum_{i=1}^{N}Degree(i)} \end{array}$$ (6)

where the *Degree*(*i*) represent the *ith* node’s degree and *N* is the total number of the nodes in the network.

## 3 Correlation of multiplex network structure information entropy and overlapping links

### 3.1 Structure entropy of multiplex network

Entropy, introduced from the concept of thermodynamics, is a measure to describe the disorder degree of a system. The more uniform the heat energy distribution, the greater the entropy. In this paper, structure entropy is used to measure the correlation between combined layers in multiplex networks. For any combined layer, the greater its entropy, the stronger the correlation between layers, and thus the more stable it is to some extent. For scale-free networks [34], due to the severe heterogeneity of the network structure, it is difficult for scale-free networks to maintain the stability of the system when targeted attacks are carried out on hub nodes. Analyzed from the perspective of structure entropy, the scale-free network has a power-law degree distribution, so the nodes have different probabilities of structure degree distribution, and the calculated network structure entropy is small. While for fully connected networks [35, 36], which are homogeneous networks, each node has the same connectivity distribution and the network structure exhibits the most stable state. Analyzed from the perspective of network structure entropy, each node has equal connectivity probability, the network structure entropy is the largest, and the system is the most stable.

In this paper, the application of complex network structure entropy is extended to multiplex networks, which is used to measure the correlation of combined layers [31, 32], and assess the stability of the network structure. Considering a multiplex formed by *N* labeled nodes *i* = 1, 2, …, *N* and *M* layers, we define the structure entropy of the multiplex network as,

EM=-∑i⊆Npilog(pi2|X|-1ε∑i⊆Nα≠β⊆Mmiα∩miβmiα∪miβ),

$$\begin{array}{c} p_{i}=\frac{k_{i}}{\sum_{i\subseteq N}k_{i}},\left(0<\varepsilon <1\right) \end{array}$$ (7)

where *k*_*i*_ is expressed as the degree of node *i* in the overlapping links mapping network, |*X*| is the cardinality of multidegree node *i*, $m_{i}^{\alpha }\cap m_{i}^{\beta }$ represents the overlapping edge set between network nodes at different layers, and $m_{i}^{\alpha }\cup m_{i}^{\beta }$ represents the set of connected edges of all nodes in different network layers.

Taking the two-layer multiplex network in Fig 2 as an example. The two-layer network in the figure overlaps two edges and connects three nodes. Mapping the two-layer network to one layer, and the degrees of node 1, node 2 and node 4 are respectively 1, 3, and 3, then |*x*| = 3. In this paper, for the convenience of calculation, *ε* is taken as 0.5. The structure entropy associated with overlapping links at different layers is calculated as follows.

$$\begin{array}{c} E_{1,2}\;=\;-\frac{1}{7}\;\text{log}\left(\;\frac{\frac{1}{7}}{2^{3}-1}0.5^{\frac{2}{6}}\;\right)\;-\;\frac{3}{7}\;\text{log}\left(\;\frac{\frac{3}{7}}{2^{3}-1}0.5^{\frac{2}{6}}\;\right)\;-\;\frac{3}{7}\;\text{log}\left(\;\frac{\frac{3}{7}}{2^{3}-1}0.5^{\frac{2}{6}}\;\right)\\ \;=4.5895 \end{array}$$

### 3.2 Numerical simulation

**Fig 3:**
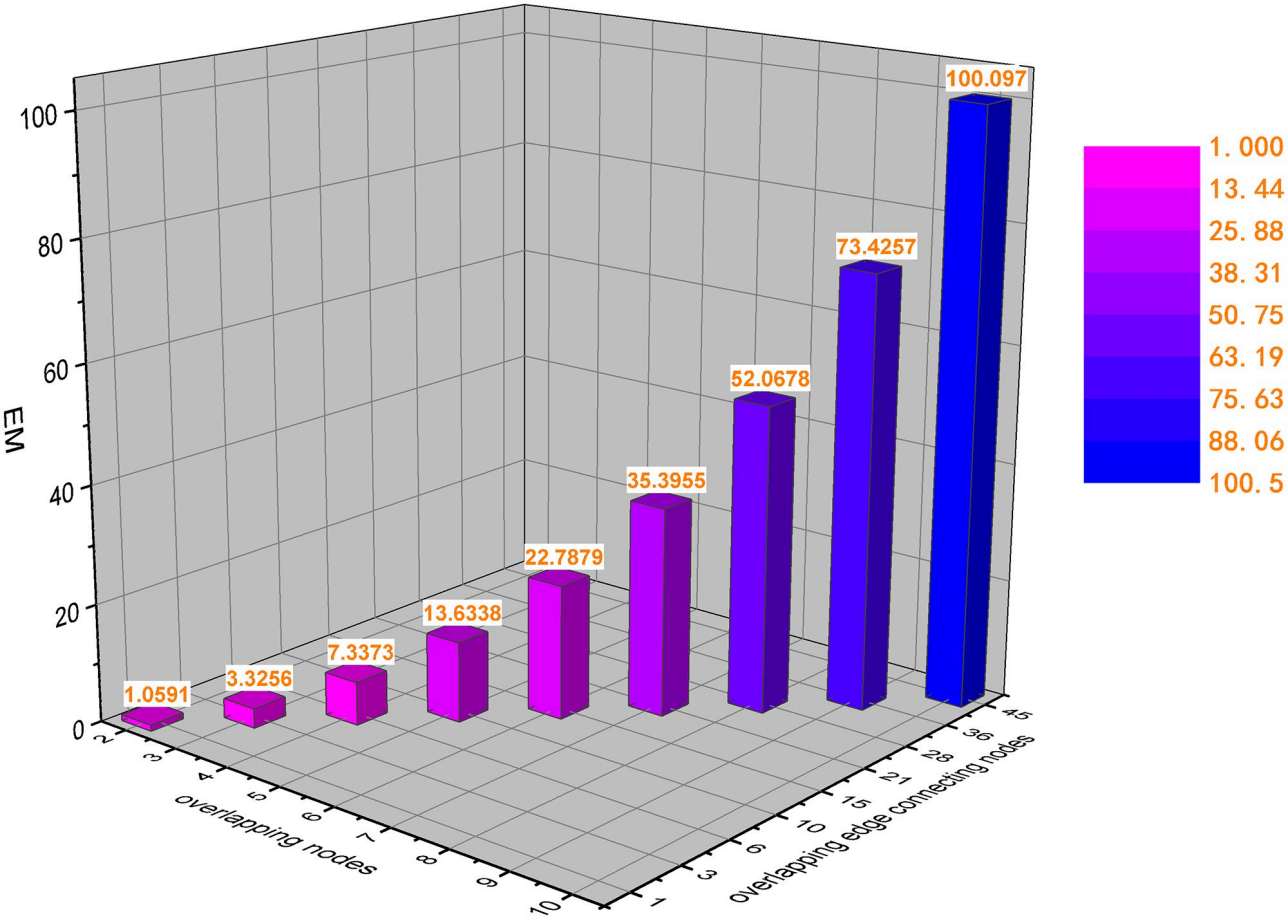
Three-dimensional coordinate histogram of the change of structure information entropy by overlapping edges and overlapping edge connected nodes. The X-axis, Y-axis, and Z-axis represent the number of overlapping edges, the number of overlapping edge connected nodes, and the network structure entropy of the combined layer of the multiplex network, respectively.

To illustrate the correlation between the structure entropy in this paper and the overlapping links of the combined layers in multiplex network, we construct a two-layer multiplex network with *N* = 10 and *M* = 2. To avoid the randomness of node connection, set the connection between neighbor nodes to be fully connected. Table. 1 shows the calculated structure entropy with the increase of overlapping edges in the multiplex network. In Table 1, with the increasing number of overlapping edges and overlapping edge connected nodes, the network structure entropy also increases. Fig 3 is a visualization of the data in Table 1.

**Table 1:** Structure entropy of different overlapping edges and overlapping edge connected nodes.

Overlapping edge connection nodes	2	3	4	5	6	7	8	9	10
Overlapping edges	1	3	6	10	15	21	28	36	45
*E*_*M*_	1.0591	3.3256	7.3373	13.6338	22.7879	35.3955	52.0678	73.4245	100.0970

According to the results shown, the greater the structure entropy, the more similar the two-layer network structure is. When the two-layer network structure is the same, that is, all the edges overlap completely, the multiplex network can be mapped into a single-layer weighted network. In Table 1, when the number of overlapping nodes is 10 and the number of overlapping edges is 45, the network is equivalent to a single-layer fully connected weighted network. At this time, the structure entropy is the largest and the stability of the system is the strongest. Therefore, we speculate that the more overlapping parts between layers in a multiplex network, the stronger the correlation between layers. Moreover, as the number of network layers increases, the more difficult it is to maintain a steady state.

## 4 Application of network structure information entropy in actual multiplex network

To verify the above conjecture, four different types of actual multiplex networks are tested. The validity of the proposed method is confirmed by analyzing the relationship between the test results and the network performance behavior. The node connection data of these four real multiplex networks are shown in Table 2.

**Table 2:** Four actual multiplex network node connection data.

Multiplex networks	Layers	Nodes	Edges	Average degree	Average weight degree
AUCS [37]	5	61	620	11.574	20.328
Tailor shop [38]	4	39	1018	15.282	26.103
C. elegans [39]	3	279	5863	16.405	21.014
7thGraders [40]	3	29	740	12.759	20.414

### 4.1 AUCS multiplex network

The data collection for the AUCS multiplex network was conducted among the employees of the Department of Computer Science at Aarhus University [37]. The population of the study is 61 employees (out of the total number of 142) who decided to join the survey, including professors, postdoctoral researchers, PhD students and administration staff. The multiplex social network was composed of five online and offline relationships (Facebook, Leisure, Work, co-authorship, Lunch) between these employees. There are 61 nodes in total, with 620 edges. The first to fifth layers correspond to the five relationships of work, facebook, co-authorship, leisure, lunch. The co-authorship network is the smallest and less connected of all layers, the work and lunch networks have the most edges and the two layers are most closely connected, and the Facebook network has the highest average node degree.

Using Eq 7 to calculate the structure entropy between each group of combined layers for the AUCS multiplex networks. For the five-layer multiplex network, there are $C_{5}^{2}+C_{5}^{3}+C_{5}^{4}+C_{5}^{5}=26$ combination results between different layers. The calculation results are shown in Table 3.

**Table 3:** Structure entropy of overlapping links between layers of aucs multiplex network.

Layers	Overlapping edges	Overlapping edge connected nodes	*E*_*M*_
*L*_1,2_	**48**	**30**	**34.6471**
*L*_1,3_	13	19	23.1166
*L*_1,4_	61	42	47.0144
*L*_1,5_	98	55	60.2823
***L*_2,3_**	**8**	**11**	**14.4433**
*L*_2,4_	29	24	28.3007
*L*_2,5_	50	30	34.8224
*L*_3,4_	10	16	19.6088
*L*_3,5_	18	24	28.2379
***L*_4,5_**	**48**	**40**	**44.9142**
*L*_1,2,3_	5	10	13.2524
*L*_1,2,4_	17	20	24.2819
*L*_1,2,5_	27	21	25.3561
***L*_1,3,4_**	**8**	**13**	**16.5887**
***L*_1,3,5_**	**11**	**17**	**21.0225**
*L*_1,4,5_	40	38	43.0005
*L*_2,3,4_	4	8	10.9734
*L*_2,3,5_	7	11	14.3880
*L*_2,4,5_	14	16	19.8058
*L*_3,4,5_	9	14	17.5657
*L*_1,2,3,4_	4	8	10.9155
*L*_1,2,3,5_	4	8	10.9520
***L*_1,2,4,5_**	**11**	**15**	**18.8339**
*L*_1,3,4,5_	7	11	14.3410
*L*_2,3,4,5_	3	6	8.4995
*L*_1,2,3,4,5_	3	6	4.4855

From the results in Table 3, the network structure entropy of layer 1 and layer 5 (work network and lunch network) is the largest in the AUCS multiplex network. This shows that the structure of the work network and the lunch network among the employees of Computer Science department at Aarhus is the most similar, and the relationship between these two layers of networks is the most stable. It is also consistent with the behavior exhibited by the network itself.

**Fig 4:**
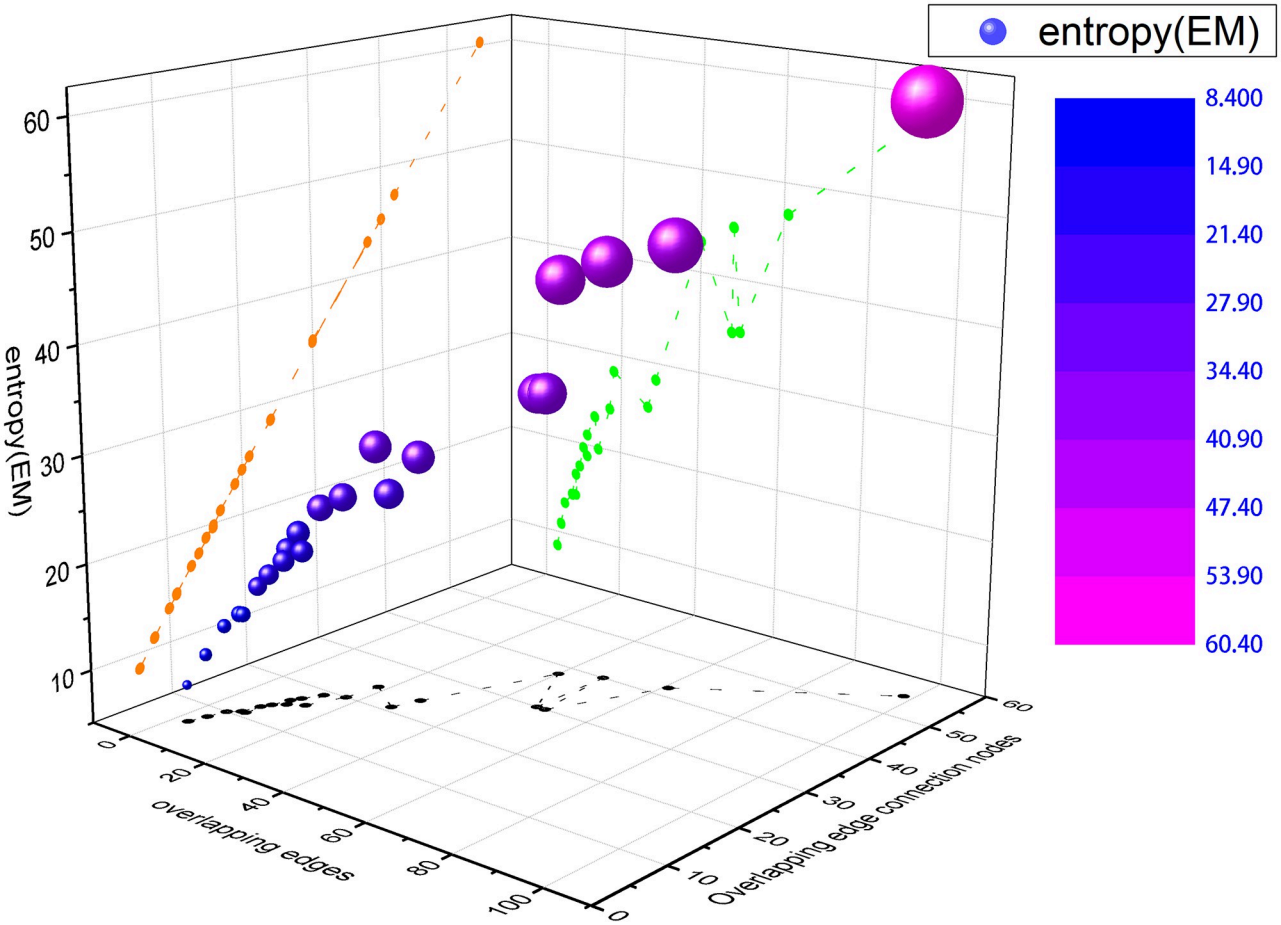
Three-dimensional coordinate scatter plot of aucs multiplex network. The green projection points reflect is the relationship between the overlapping edges and entropy of the combined layers. The orange projection points represent the relationship between the overlapping edge connected nodes and the entropy of the combined layer.

Fig 4 is a three-dimensional coordinate scatter plot constructed for the structure entropy of AUCS multiplex network. According to the trend shown in the figure, the larger the overlapping edges and the overlapping edge connected nodes are, the greater the structure entropy is. However, the conclusion of structure entropy on multiplex networks proposed in this paper does not stop there. When the overlapping edges of different network layers are equal and the overlapping edge connected nodes are different, the more overlapping edge connected nodes, the greater the structure entropy, as shown in the calculation results of layers *L*_1,2_ and *L*_4,5_ in Table 3. When the overlapping edges and overlapping edge connected nodes of different layers are equal, the more overlapping network layers, the smaller the structure entropy, such as *L*_2,3,5_ and *L*_1,3,4,5_, *L*_2,3,4,5_ and *L*_1,2,3,4,5_ in Table 3. When the number of layers, overlapping edges and overlapping edge connected nodes of different layer networks are all equal, the method in this paper can still distinguish which layer networks are the most similar and the most stable in structure, such as *L*_2,3,4_, *L*_1,2,3,4_ and *L*_1,2,3,5_ in Table 3.

### 4.2 Tailor shop multiplex network

The second practical example taken was a ten-month interaction in a tailor shop in Zambia (then Northern Rhodesia) [38]. This is a four-layer multiplex network. Layers represent two different types of interactions, recorded at two different times (seven months apart) over a period of one month. Layer 1 and layer 2 were “instrumental” (work and assistance-related) interactions at the two times; layer 3 and layer 4 recorded “sociational” (friendship, socioemotional) interactions. The data are particularly interesting since an abortive strike occurred after the first set of observations, and a successful strike took place after the second.

For tailor shop four-layer multiplex network, there are $C_{4}^{2}+C_{4}^{3}+C_{4}^{4}=11$ combination results between different layers. The results of calculating the network structure entropy between the combined layers are shown in Table 4.

**Table 4:** Structure entropy of overlapping links between layers of tailor shop multiplex network.

Layers	Overlapping edges	Overlapping edge connected nodes	*E*_*M*_
*L*_1,2_	45	25	29.4616
*L*_1,3_	116	34	39.1831
*L*_1,4_	74	29	33.9143
*L*_2,3_	76	31	35.9806
*L*_2,4_	91	33	37.5822
*L*_3,4_	206	37	41.7379
*L*_1,2,3_	44	24	28.4273
*L*_1,2,4_	45	25	29.4372
*L*_1,3,4_	68	27	31.5439
*L*_2,3,4_	73	30	34.9191
*L*_1,2,3,4_	4	8	10.9155

**Fig 5:**
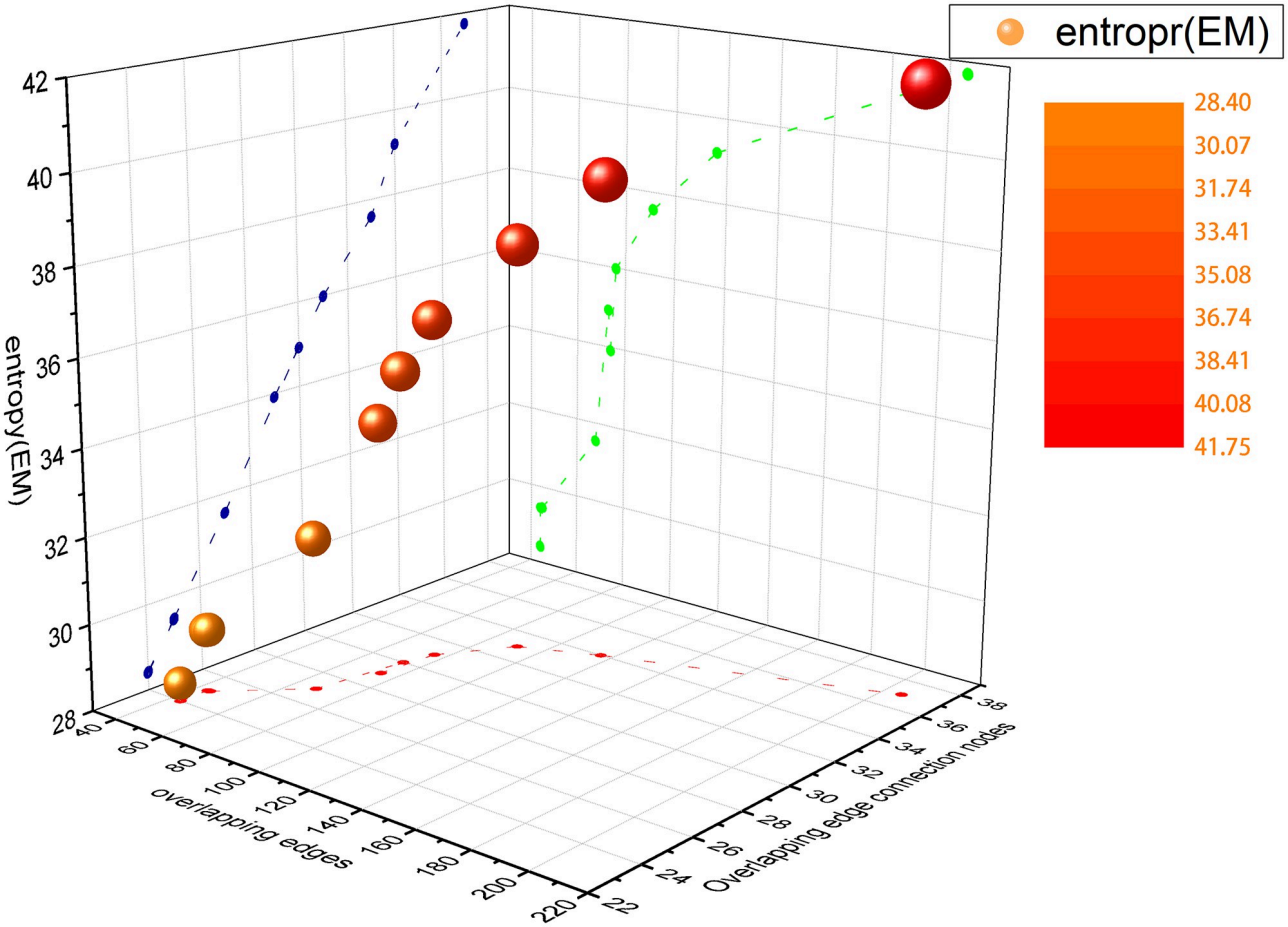
Three-dimensional coordinate scatter plot of tailor shop multiplex network. The green scatter is the trend of the structure entropy about the overlapping edges. The blue scatter is the change in structure entropy with relation to the overlapping edge connected nodes. The red scatter is the correspondence between the overlapping edges and the overlapping edge connected nodes.

In Table 4, the entropy of layers 3 and 4 are the largest, indicating that this tailor shop has the most frequent friendships and emotional interactions during the ten-months recorded. And after this set of interactions a successful strike occurred. However, the structure entropy of layer 1 and layer 2 is the smallest among all two-layer overlapping networks, indicating that the interaction between the two layers is unstable, so this strike is a failure. This also proves that the proposed entropy can effectively measure the correlation between different layers, and the larger the entropy is, the more similar the structures between different layers will be, the greater the correlation will be. Fig 5 is a three-dimensional coordinate scatter visualization of Table 4. The figure shows that the structure entropy of the network is positively correlated with the overlapping edges and the overlapping edge connected nodes.

### 4.3 C. elegans multiplex network

The C. elegans multiplex network representing the multiplex neuronal network of the nematode “Caenorhabditis Elegans” [39]. Caenorhabditis elegans connectome, where the multiplex consists of layers corresponding to different synaptic junctions: electric (“ElectrJ”), chemical monadic (“MonoSyn”), and polyadic (“PolySyn”).

**Fig 6:**
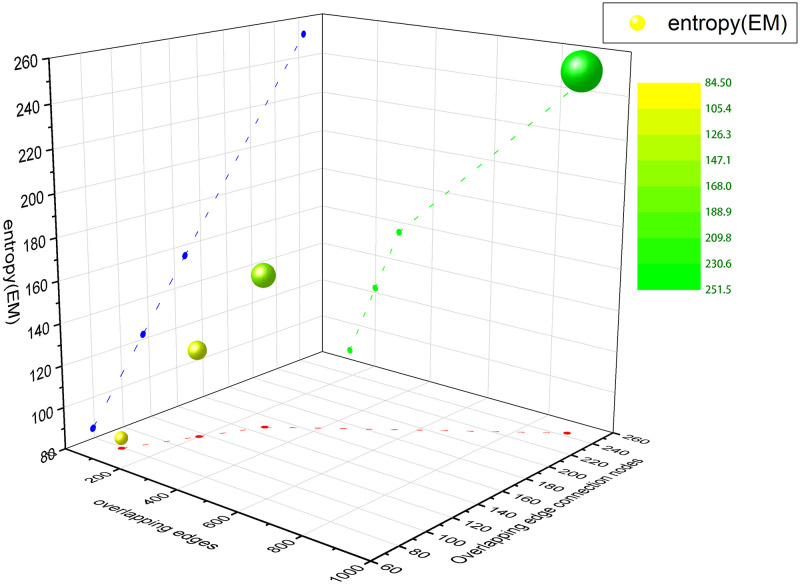
Three-dimensional coordinate scatter plot of C. elegans multiplex network. The green scatter shows that the structure entropy has a positive trend about the overlapping edges. The blue scatter shows that the structure entropy is linearly increasing with respect to the overlapping edge connected nodes.

For the C. elegans multiplex network, there are $C_{3}^{2}+C_{3}^{3}=4$ combination results between different layers. The calculation results are shown in Table 5 and the visualization is shown in Fig 6.

**Table 5:** Structure entropy of overlapping links between layers of C. elegans multiplex network.

Layers	Overlapping edges	Overlapping edge connected nodes	*E*_*M*_
*L*_1,2_	199	116	122.3995
*L*_1,3_	283	148	154.7746
*L*_2,3_	910	244	251.3750
*L*_1,2,3_	68	27	84.9517

From the results in Table 5, the structure entropy of layer 2 and layer 3 is the largest, indicating that the neuronal synaptic connection structures between chemical monadic (“MonoSyn”) layer and polyadic (“PolySyn”) layer are the most similar, and the chemical monadic and chemical polyadic layers interact more closely with each other than with electric layer in the C. elegans multiplex networks. From Fig 6, the more overlapping edges and overlapping edge connected nodes in different network layers in the C. elegans multiplex network, the greater the structure entropy.

### 4.4 7thGraders multiplex network

The 7thGraders multiplex network data were collected by Vickers from 29 seventh-grade students in a school in Victoria, Australia. Students were asked to nominate their classmates on several relations including the following three (layers):

1. Who do you get on with in the class?
2. Who are your best friends in the class?
3. Who would you prefer to work with?

The interactions between layers represent these three types of relationships.

**Fig 7:**
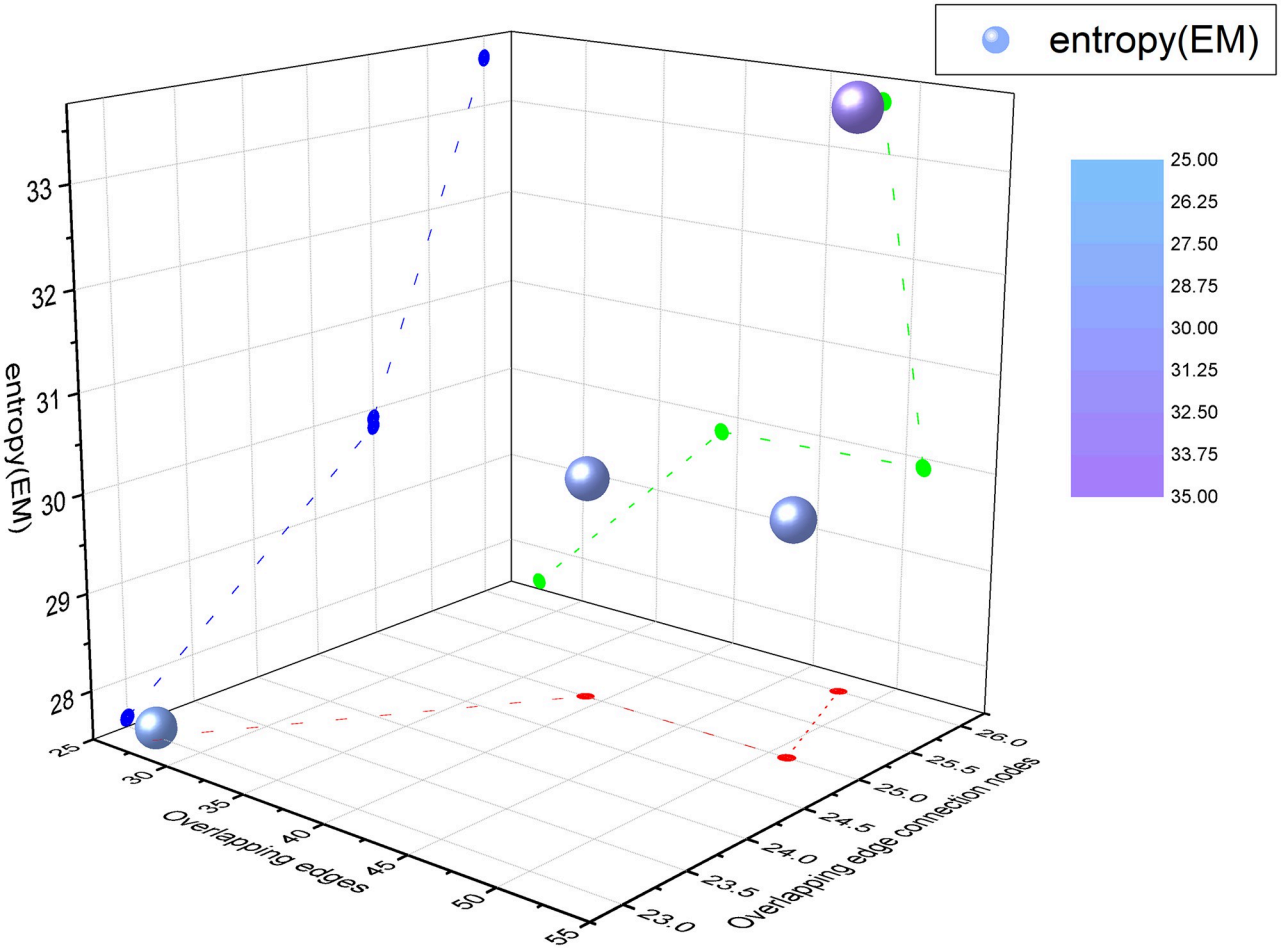
Three-dimensional coordinate scatter plot of 7thGraders multiplex network. The green scatter shows that the structure entropy varies irregularly about the overlapping edges. The blue scatter shows that the structure entropy is positively correlated with the overlapping edge connected nodes.

For the 7thGraders multiplex network, there are $C_{3}^{2}+C_{3}^{3}=4$ combination results between different layers. The calculation results are shown in Table 6 and the visualization is shown in Fig 7.

**Table 6:** Structure entropy of overlapping links between layers of 7thGraders multiplex network.

Layers	Overlapping edges	Overlapping edge connected nodes	*E*_*M*_
*L*_1,2_	39	25	29.8104
*L*_1,3_	48	26	33.5137
*L*_2,3_	51	25	29.9011
*L*_1,2,3_	27	23	27.5925

From Table 6, the structure entropy of the interaction between layer 1 and layer 3 is the largest, and the structure entropy of the overall interaction among the three layers is the smallest. This indicates that most of these 27 students in the class who are willing to get on with each other also prefer to work together. From Fig 7, the proposed entropy is related to the overlapping edges and overlapping edge connected nodes of the combined layer of the multiplex network, but it is not a linear growing relationship with the overlapping edges and the overlapping edge connected nodes.

The trends of these four real network structural entropy scatter plots and the mapping relationships of the scatter points on each coordinate plane all reflect the same rule, that is, the more overlapping edges and overlapping edge connected nodes between the combined layers of the network, the higher the entropy. However, the proposed structure entropy is not linearly related to the overlapping edges and overlapping edge connected nodes, instead, it is also related to the structure degree distribution of the overlapping part. The structure entropy of the network is the largest and the network structure is the most stable when the network structures of different layers are the same.

According to the calculation results of the above four actual multiplex network structure entropy, the entropy of the network structure with more combined layers is smaller than that of the network structure with fewer combined layers. This also confirmed our conjecture, which is that the more the number of combined layers of the network, the more difficult it is to unify the inter-layer interactions, and the more difficult it is for the system to reach a steady state.

## 5 Conclusion

The layers of real multiplex networks are not completely independent of each other, but rather have some correlation. For example, a large degree node in a micro-blog network is often a large degree node in a WeChat network as well. This correlation between layers is associated with overlapping parts. In this paper, we propose a new network structure entropy that can be a promising metric for measuring the correlation between multiplex network layers. The proposed entropy is used for multiple real multiplex networks and the measurement results are consistent with the behavior between networks. This also demonstrates the validity and applicability of the method in this paper.

The interaction of each layer of the multiplex network will make the structure of each layer of the network evolve. In this paper, the change of the structure of each layer of the multiplex network can be inferred by the change of the correlation of each layer of the network. This also brings a new research idea to the research of multiplex network dynamics. Such as in the multiplex network of disease transmission, to help find the way of disease transmission and reduce the probability of infection. And in the protein gene and cancer cell interaction network, the successful injection of drug targets blocks the spread of cancer cells, and so on.
